# Unraveling the threads of trauma: how adverse childhood experiences shape suicidal behaviors and help-seeking attitudes in black young adults

**DOI:** 10.1186/s12889-025-24982-5

**Published:** 2025-10-31

**Authors:** Donte T. Boyd, Camille R. Quinn, Addie Weaver, Ed-Dee G. Williams, Myles I. Durkee, Elena L. Pokowitz, Danae Ross

**Affiliations:** 1 School of Social Work, The Ohio State University, 1947 N. College Road, 325K, Columbus, OH USA; 2 School of Social Work, University of Michigan, Ann Arbor, MI USA; 3 Center for Equitable Family and Community Well-Being, School of Social Work, University of Michigan, Ann Arbor, USA; 4 School of Social Work, Boston College, Chestnut Hill, MA USA; 5 Department of Psychology, University of Michigan, Ann Arbor, MI USA

**Keywords:** Suicide, Black young adults, ACEs, Mental health help-seeking attitudes, Intersectionality framework, Trauma informed approach

## Abstract

Suicide rates among Black young adults have significantly increased over the past two decades, presenting a critical public health issue. Although research indicates the association of positive mental health attitudes with better outcomes, limited attention has been paid to the structural and cultural factors that influence suicidal behaviors and help-seeking attitudes in this demographic. Guided by intersectionality and trauma-informed frameworks, this study examines how adverse childhood experiences (ACEs), age, and suicidal behaviors interact to influence mental health help-seeking attitudes among Black young adults. We recognize that these outcomes are shaped by interlocking systems of oppression, including racism, ageism, and historical mistrust of mental health institutions. The sample consisted of 359 Black young adults aged 18 to 24 (*M* = 21; *SD* = 1.90), recruited through Qualtrics panels (a pre-recruited group of individuals who have agreed to participate in surveys) in the Midwestern United States from February 1, 2023, to April 1, 2023. Using path analysis, our study results indicated that ACE scores had a significant and positive direct relationship with suicidal ideation (β = 0.29, *p* < .001) and suicide planning (β = 0.30, *p* < .001). Additionally, there was a significant indirect association with suicide attempts (β = 0.04, *p* < .001). Further, suicide attempts negatively impacted attitudes toward seeking mental health help (β = -0.14, *p* < .01). Suicidal ideation and planning also had significant negative indirect associations with these attitudes (β = -0.05, *p* = .01; β = -0.08, *p* = .01). These findings underscore the urgent need for culturally grounded and structurally responsive suicide prevention and intervention strategies that reflect the lived experiences of Black young adults. By addressing the unique interplay of trauma, identity, and systemic inequity, we can enhance mental health support and overall well-being for this population.

## Introduction

Suicide rates among Black young adults have significantly increased over the past 15 years, representing a public health issue requiring urgent attention and action. Research demonstrates a 37% increase in suicide rates among Black young adults (aged 18 to 24) from 2018 to 2021 [1–3]. Similarly, concerning increases in suicide attempts and nonfatal suicidal behaviors among Black young adults have also been observed [4–6]. Identifying factors that drive suicidal ideation, planning, and nonfatal suicide attempts among Black young adults is imperative to inform culturally relevant prevention and intervention strategies aimed at reducing suicide rates and improving mental health outcomes in this population.

Existing research on suicidality among Black young adults indicates that systemic racism, socioeconomic disparities, mental health stigma, and disparities in access to mental health treatment—including limited access to culturally competent services—are associated with the rise in suicidal behaviors among Black young adults aged 18 to 24 [1, 3, 7]. However, more research is needed to better understand the sociodemographic factors, including adverse childhood experiences (ACEs) and age, that may relate to suicidal behaviors. Further, there is limited research on the relationship between suicidality and mental health help-seeking attitudes among Black young adults.

Literature consistently shows the negative impact of ACEs on mental health outcomes [8–10], especially with adolescents. Individuals with higher ACE scores are more likely to experience post-traumatic stress disorder, depression, and anxiety, and are at an increased risk of suicidality compared to peers with lower ACE scores [11, 12]. Further, ACEs have been identified as a major risk factor for future suicidal behaviors [13, 14]. Despite strong evidence demonstrating the negative relationship between ACEs and mental health, as well as research suggesting that Black young adults are more likely than their White counterparts to experience ACEs [15–17], much of the existing literature has not explicitly focused on Black young adults in examining this relationship.

Additionally, research suggests that age during the period of emerging adulthood may relate to suicidality, as there may be within-group age differences among young adults ages 18 to 24 that affect suicide rates. Studies have shown that suicidal behaviors among Black young adults (aged 20 to 21 and 22 to 23) have increased by 68% and 55%, respectively, whereas suicidal behaviors have risen by 46% among Black young adults aged 18 to 19 and by 29% among those aged 24 to 25 [1, 6]. This suggests that suicide risk may vary among emerging Black adults, requiring age-based targeted prevention and intervention approaches; also needed is additional research focused on the relationship between age and suicidality among Black young adults.

Moreover, Black young adults’ attitudes concerning mental health are possibly shaped by cultural, historical, and social experiences unique to their communities [18]. Existing studies indicate that Black young adults experience higher rates of untreated mental disorders and lower rates of mental health service participation than their white peers [19–21]. Lower participation in mental health services may relate to Black young ’ attitudes and beliefs about mental health and help-seeking. For instance, Williams et al. [21] found that Black adolescents with strong attitudes of self-reliance—believing they should manage their mental health needs independently—were 77% less likely to use resources available in their schools compared to peers who did not hold such strong beliefs. However, research focused on mental health help-seeking attitudes among Black young adults ages 18 to 24 is lacking, as also the literature examining the relationship between suicidality and mental health help-seeking attitudes. Understanding Black young adults’ attitudes toward mental health and help-seeking, as well as examining the relationship between suicidality and mental health help-seeking attitudes, is crucial for reducing treatment access disparities, developing and tailoring prevention and intervention strategies accessible and acceptable to Black young adults, and promoting this population’s overall well-being.

Despite documented increases in suicide rates and suicidal behaviors among Black young adults, limited research has focused on the factors driving suicidality in this population. Further, the relationship between suicidality and attitudes toward mental health help-seeking has been understudied. Identifying the factors affecting suicidality among Black young adults is critical for informing prevention and intervention efforts, with scholars specifically calling for further investigation into the factors associated with planning to die by suicide among this group [22]. This study addresses current gaps in the literature by examining the relationships between ACEs, age, suicidal ideation, planning, attempts, and mental health help-seeking attitudes among Black young adults. Besides deepening our understanding of factors potentially related to suicidality, this study encompasses three aspects thereof —ideation, planning, and attempts—providing a more comprehensive understanding of the relationship between ACEs, age, suicidality, and mental health help-seeking attitudes.

### Theoretical frameworks

This paper explores how intersectionality and trauma-informed frameworks illuminate the relationships between ACEs, age, suicide risk, and mental health help-seeking attitudes among Black young adults [22–25]. The intersectionality theoretical framework posits that interlocking systems of oppression—such as racism, sexism, classism, and heteronormativity—shape overlapping social identities, including race, gender, socioeconomic status, and sexual orientation [26]. For Black young adults, these intersecting identities produce unique experiences of marginalization, structural exclusion, and psychological distress. This lens enables us to interpret help-seeking attitudes not as isolated individual choices, but as deeply contextual responses to historical and ongoing systemic inequities that have eroded trust in mental health institutions and limited access to culturally affirming care [26]. By situating our findings within this framework, we underscore the need for mental health interventions to not only be culturally tailored, but also structurally responsive to the compounded vulnerabilities faced by this population.

Complementing this, trauma-informed frameworks [23–25] emphasize the pervasive and cumulative impact of trauma on individuals and communities. This approach recognizes that trauma is not only personal but also collective and systemic, rooted in historical injustices, institutional neglect, and socioeconomic disadvantage. For Black young adults, exposure to ACEs often occurs in environments shaped by racialized surveillance, economic instability, and community-level stressors. A trauma-informed lens urges practitioners and researchers to understand these experiences in a broader social and historical perspective, acknowledging how trauma disrupts development, impairs coping, and elevates suicide risk [24]. Together, these frameworks provide a comprehensive understanding of Black young adults’ layered vulnerabilities, systemic inequalities, and historical traumas, affirming the need for equity-driven approaches to mental health care and suicide prevention.

Research indicates that ACEs are critical predictors of suicidal thoughts and behaviors, as they can lead to long-term psychological distress and dysfunction. Black youth, who disproportionately experience ACEs due to systemic inequities, are at an increased risk for suicidality [27, 28]. Moreover, the intersection of ACEs with age-related factors reveals that younger individuals may experience these traumas differently, often leading to delayed or intensified mental health crises. The relationship between ACEs, age, and suicide risk also influences mental health help-seeking attitudes. Many Black young adults encounter barriers in accessing mental health services, stemming from stigma, historical mistrust of healthcare systems, and cultural expectations to remain resilient [29, 30]. This reluctance to seek help can be compounded by the internalization of the “Strong Black Woman” stereotypes, which discourage vulnerability and promote self-silencing [31].

Understanding these dynamics is critical for developing culturally responsive interventions that address the lived experiences of Black young adults. Integrating intersectionality and trauma-informed frameworks allows us to craft strategies that not only promote mental health help-seeking attitudes but also confront the root causes of suicidality within this population. The intersection of ACEs, age, and systemic trauma provides a comprehensive lens for assessing suicide risk and Black young adults’ attitudes toward mental health care. By centering these frameworks, we can better address the complexities of their experiences and promote resilience and recovery in this vulnerable population.

Suicide risk is also posited to influence mental health help-seeking attitudes [32]. Many Black young adults encounter barriers to mental health care, including stigma and mistrust of healthcare systems [33]. For instance, a 23-year-old Black woman may refrain from seeking help due to concerns about being misunderstood or judged by providers. Cultural narratives, such as the “Strong Black Woman” stereotype, further exacerbate these barriers by discouraging vulnerability [34].

### Current study

This study employs path analysis to explore the intricate associations between ACEs, age, suicide risk, and mental health help-seeking attitudes among Black young adults. Using intersectionality and trauma-informed frameworks, our research aims to deepen the understanding of how systemic factors shape individual experiences and inform targeted interventions that promote resilience and enhance mental health outcomes for this population. By examining the relationships among ACEs, age, and three dimensions of suicidality—ideation, planning, and attempts—this study addresses significant gaps in the literature and provides a comprehensive perspective on the factors influencing suicidality and help-seeking behaviors among Black young adults.

We hypothesize as follows:

- H_1_: Age does not have a significant direct effect on planning to die by suicide, suggesting that the propensity for such planning may be consistent across different age groups.
- H_2_: The relationship between ACEs and mental health help-seeking attitudes is mediated by suicidal ideation and suicide attempts, such that higher ACEs lead to increased suicidal ideation and attempts, which, in turn, decrease help-seeking attitudes.
- H_3_: The relationship between ACEs and suicide attempts is partially mediated by planning to die by suicide, indicating that higher ACEs contribute to more planning behaviors, which, in turn, lead to increased attempts.
- H_4_: There is a negative relationship between suicidal ideation and suicide attempts, which negatively influences mental health help-seeking attitudes, suggesting that individuals who experience these behaviors are less inclined to seek help.

These hypotheses can guide further research and investigation.

## Methods

### Procedures

The sample for this study comprised 359 Black young adults aged 18 to 24 living in the Midwest United States. Participants were recruited through Qualtrics panels, an online survey delivery service renowned for its effectiveness in reaching hard-to-engage populations [1, 3]. Qualtrics panels consist of individuals who have agreed to participate in surveys regularly for research purposes. Before individuals were admitted to the consumer panel, their names, addresses, and dates of birth underwent verification through third-party validation processes. Panel members received email invitations or prompts on the survey platform to participate in specific surveys. These invitations contained a hyperlink to access the survey and outlined any available incentives. The research goals were also explained to the participants.

Prior to participating in the survey, all participants were required to give their consent. The survey achieved an 85% response rate. Individuals were eligible to participate if they self-identified as Black, were aged 18 to 24, and resided in a Midwestern state (e.g., Ohio, Michigan, or Illinois). The online survey took approximately 20 min to complete and included questions about mental health and services, experiences of racism, and sexual health. The Institutional Review Board at The Ohio State University approved all protocols for this study, which was conducted in accordance with ethical standards for research involving human subjects. Informed consent was obtained from all participants prior to their participation in the study. This commitment to ethical standards is paramount in safeguarding the rights and welfare of our participants.

### Measures

#### Independent variables

*Adverse Childhood Experiences (ACEs).* ACEs, defined per terms from the Centers for Disease Control and Prevention (2023), were identified as exposure to physical, sexual, or emotional abuse, neglect, parental incarceration, alcoholism, divorce, or separation, poverty, community violence, and/or foster home placement before the age of 18 [4, 35]. Each exposure was coded as a binary (*yes*/*no*) variable, and exposures were summed to create a cumulative ACE score ranging from zero (unexposed to any ACE) to 10 (exposed to all 10 ACEs). Respondents who scored 4 or above were considered to have a high ACE score [35, 36].

*Age.* Age was measured as a continuous variable ranging from 18 to 24.

#### Mediating variables: past-year suicide ideation, planning, or attempt

Three separate variables developed by Goodwill et al. [37] were used to assess past-year suicidal ideation, suicide planning, and suicide attempts. The variable for past-year suicidal ideation was measured using a single dichotomous item that asked respondents whether they had considered ending their life in the previous 12 months. The response categories were 1 = *yes* and 0 = *no* [37]. The variable for past-year suicide planning was measured using a single dichotomous item that asked respondents whether they had planned to end their life within the previous 12 months. The response categories were 1 = *yes* and 0 = *no* [37]. The variable for past-year suicide attempts was measured through a single dichotomous item that asked respondents to indicate whether they had attempted to end their life within the previous 12 months. The response categories were 1 = *yes* and 0 = *no* [27].

#### Dependent variable: mental help-seeking attitudes scale

The Mental Help-Seeking Attitudes Scale (MHSAS) assesses attitudes toward seeking help from mental health professionals, consists of nine items, and employs a 7-point semantic differential scale, with items 2, 5, 6, 8, and 9 reverse-coded. Scores, ranging from 1 to 7, are calculated by summing item scores and dividing by the total number of items answered. Higher scores indicate more favorable attitudes toward seeking help from mental health professionals. The Mental Help-Seeking Attitudes Scale has demonstrated good reliability, with a Cronbach’s alpha value of 0.92 [38]. In the present study, Cronbach’s alpha was 0.90.

*Demographic variables* were also collected, including participants’ educational attainment, sex at birth, and total household income. These variables served as demographic covariates in the analyses.

### Statistical analysis

All data were checked for missing values; no missing data were found among the primary analytic variables and covariates. Descriptive statistics were calculated (see Table 1), along with bivariate analyses to assess reliability estimates (Cronbach’s alpha). Table 2 presents correlation analyses between the key study variables.

**Table 1:** Demographics of study participants (*N* = 359)

Demographic Variable	*N*	%	M	SD
Age (range: 18 to 24)			21.22	1.90
Adverse Childhood Experiences (range: birth to 10)			5.00	2.41
Mental Health-Seeking Attitudes (range: 1 to 5)			4.37	0.76
Race and Ethnicity				
African American	290	81		
Afro-Caribbean/West Indian	35	10		
African	21	6		
Afro-Latino	10	3		
Gender				
Male	84	23		
Female	254	71		
Transgender Female	2	0.01		
Transgender Male	3	0.01		
Nonbinary	14	4		
Other	2	0.01		
Sex Assigned at Birth				
Male	90	23		
Female	269	77		
Education Completed				
No High School	30	8		
High School Diploma or GED	144	41		
Currently in School	32	9		
Some College, Associate Degree, or Trade School	114	32		
College, Postgraduate, or Professional Degree	24	7		
Employment Status				
Homemaker	15	4		
Full Time	104	29		
Part Time	119	34		
Not Employed	74	21		
Self-Employed	28	8		
Disabled	8	2		
Other	6	2		
Income				
Low (≤ $39,999)	231	69		
Medium ($40,000–$99,999)	81	24		
High (≥ $100,000)	22	7		
Missing	20	6		
Past-Year Suicide Ideation				
Yes	179	51		
No	175	49		
Past-Year Planning to Die by suicide				
Yes	124	35		
No	230	65		
Past-Year Suicide Attempt				
Yes	87	25		
No	267	75		

**Table 2:** Bivariate correlation of study variables on mental health Help-Seeking attitudes (*N* = 359)

Mental Health Help-Seeking Attitude	1					
ACE Scores	−0.10	1				
Age	0.07	−0.03	1			
Suicide Attempts	−0.14^***^	0.26^***^	−0.10^**^	1		
Planning to Die by Suicide	−0.13^**^	0.30^***^	0.00	0.52^***^	1	
Suicide Ideation	−0.13^**^	0.29^***^	−0.11^**^	0.46^***^	0.57^***^	1

Pearson correlation coefficients are shown. p < .01 (**), p < .001 (***). Higher scores on the Mental Health Help-Seeking Attitude scale indicate more positive attitudes toward seeking help for mental health concerns. *ACE* Adverse Childhood Experiences

**Fig. 1:**
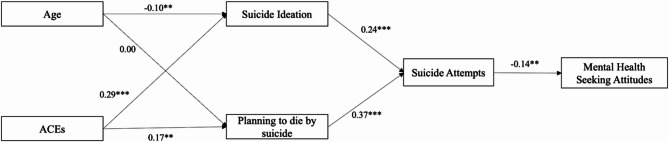
Direct and Indirect Effects of Adverse Childhood Experiences and Age on Mental Health Help-Seeking Attitudes Through Suicidal Behaviors (N = 359). Note. p < .05*, p < .01**, p < .001***. Standardized betas are reported. Models were adjusted for sex at birth, household income, and educational attainment

Our team used M-plus version 8.7 to conduct a path analysis examining the relationships among ACE scores, age, suicidal ideation, planning to die by suicide, suicide attempts, and mental health help-seeking attitudes among Black young adults (see Fig. 1). We analyzed the direct relationships of ACE scores and age with suicidal ideation and planning, as well as the direct connections of suicidal ideation and planning with suicide attempts, and suicide attempts with mental health help-seeking attitudes (see Table 3). Additionally, we explored the indirect relationships of ACE scores and age with mental health help-seeking attitudes through suicidal behaviors, and the indirect relationships of ACE scores, age, suicidal ideation, and planning to suicide attempts (see Table 4). Each model was adjusted for the covariates of participant age, college enrollment, and household income. Data were assessed to ensure that the assumptions of path analysis were met.

**Table 3:** Direct effects of adverse childhood experiences and age on mental health Help-Seeking attitudes through suicidal behaviors (*N* = 359)

Coefficient	Beta	Standardized Beta	Standard Error	95% CI
Suicide Attempts:				
Suicide Ideation◊	0.21	0.24^***^	0.05	0.12, 0.30
Planning to Die by Suicide◊	0.33	0.37^***^	0.05	0.24, 0.44
Mental Health Help-Seeking Attitudes:				
Suicide Attempts◊	−0.25	−0.14^**^	0.09	−0.43, − 0.07
Suicide Ideation:				
Age◊	−0.03	−0.10^**^	0.01	−0.05, 0.00
ACEs scores◊	−0.06	0.29^***^	0.01	0.04, 0.08
Planning to Die by Suicide:				
Age◊	0.00	0.00	0.01	−0.02, 0.03
ACEs scores◊	0.06	0.30^***^	0.01	0.04, 0.08

Unstandardized Beta and standardized betas regression coefficients are presented with standard errors and 95% confidence intervals. The model adjusted for the following covariates: sex at birth, household income, and educational attainment. p < .01**, p < .001***

*CI* confidence intervals, *ACEs* Adverse Childhood Experiences

Betas are unstandardized. Higher scores on suicide-related variables indicate greater severity or frequency of suicidal thoughts and behaviors. Higher Mental Health Help-Seeking Attitude scores reflect more positive attitudes toward seeking mental health support.

**Table 4:** (Indirect Effects of Adverse Childhood Experiences and Age on Mental Health Help-Seeking Attitudes Through Suicidal Behaviors (N = 359)

Coefficients	Beta	Standard Error	95% CI
Suicide Attempts:			
Age◊Suicide Ideation◊	−0.01	0.01	−0.02, 0.01
ACEs Scores◊ Planning to Die by Suicide ◊	0.04^***^	0.01	0.02, 0.05
Mental Health Help-Seeking Attitudes:			
Suicide Ideation◊Suicidal Attempts◊	−0.05^*^	0.02	−0.09, − 0.01
Planning to Die by Suicide◊ Suicide Attempts◊	−0.09^*^	0.03	−0.15, − 0.02
ACEs Scores◊ Planning to Die by Suicide◊ Suicide Attempts◊	−0.01^*^	0.00	−0.00, − 0.00
Age◊Suicide Ideation◊Suicide Attempts◊	0.00	0.00	−0.01, − 0.00

Reported values represent indirect effects of adverse childhood experiences (ACEs) and age on mental health help-seeking attitudes through suicidal behaviors. Models were adjusted for sex at birth, household income, and educational attainment. *p *< .05 * *p* < .01**, *p* < .001***

*CI* confidence intervals

^*^,^**^, Note. Betas are unstandardized

Model fit was considered good if the χ^2^ value was nonsignificant, the comparative fit index (CFI) and Tucker-Lewis Index (TLI) were ≥ 0.95 (adequate if ≥ 0.90), and the root mean square error of approximation (RMSEA) was ≤ 0.06 (adequate if ≤ 0.08). The Akaike information criterion (AIC) and Bayesian information criterion (BIC) were used to compare fit between models. We also included standardized beta coefficients and *p*-values to examine the associations among study variables.

## Results

### Descriptive statistics

Our sample included 359 self-identified Black young adults aged 18 to 24 (*M* = 21; *SD* = 1.90). Participants identified as African American (81%), Afro-Caribbean/West Indian (10%), continental African (6%), or Afro-Latino (3%). Almost one-quarter of the sample self-identified as male (*n* = 84; 23%), while 71% (*n* = 254) identified as female, and 4% (*n* = 21) as trans-women, trans-men, nonbinary, or other. Approximately 70% (*n* = 251) of participants reported being employed, with 29% (*n* = 104) indicating full-time employment, 34% (*n* = 122) part-time employment, and 8% (*n* = 29) self-employment. Just over 40% of the sample reported having a high school diploma or equivalent (41%; *n* = 147), while 32% (*n* = 115) reported having some college education, an associate’s degree, or trade school education. Of the participants, 57.06% reported being enrolled in or attending college, while 42.94% reported not being enrolled. Additionally, more than two-thirds of the sample (69%; *n* = 248) reported annual household incomes of $39,999 or less. Almost two-thirds of participants lived in Ohio (28%; *n* = 101), Illinois (20%; *n* = 72), or Michigan (15%; *n* = 54).

Concerning suicidality in the past year, approximately 50% (*n* = 179) of the sample reported having thought about suicide, 35% (*n* = 124) indicated having planned to end their life, and 25% (*n* = 87) reported having attempted suicide. The average ACE score for Black young adults in this sample was 5 (*SD* = 2.4). On average, this sample reported neutral attitudes toward mental health help-seeking (*M* = 4.37; *SD* = 0.76) (see Table 1).

### Bivariate correlations

Bivariate correlations of key study variables (see Table 2) revealed significant relationships between sociodemographic characteristics and suicidal behaviors. ACEs were correlated positively with suicidal ideation (*r* =.28, *p* <.001) and negatively with planning to die by suicide (*r* = −.30, *p* <.001). Age was negatively correlated with suicidal ideation (*r* = −.11, *p* <.01) and suicide attempts (*r* =.25, *p* <.001). Suicidal ideation was positively correlated with planning to die by suicide (*r* =.57, *p* <.01), and planning to die by suicide was positively correlated with suicide attempts (*r* =.51, *p* <.001). Suicidal ideation (*r* = −.13, *p* <.01), planning to die by suicide (*r* = −.13, *p* <.01), and suicide attempts (*r* = −.14, *p* <.05) were negatively correlated with mental health help-seeking attitudes.

### Path analysis

#### Direct effects

The model exhibited a good overall fit to the sample data: χ² = 10.113 (6), *p* =.12; CFI = 0.98; TLI = 0.97; RMSEA = 0.04 (95% CI = 0.000, 0.090); and AIC = 5,016.57. There were significant positive relationships between ACE scores and both suicidal ideation (β = 0.29, *p* <.001) and planning to die by suicide (β = 0.30, *p* <.001). Additionally, a significant negative relationship was observed between age and suicidal ideation (β = −0.10, *p* <.01). Suicidal ideation was significantly and positively associated with suicide attempts (β = 0.24, *p* <.001), as was planning to die by suicide (β = 0.37, *p* <.001). Finally, a significant negative relationship was observed between suicide attempts and mental health help-seeking attitudes (β = −0.14, *p* <.01) (see Table 3).

#### Indirect effects

Our model revealed that ACE scores were significantly and positively associated with suicide attempts through the mediating variables of planning to die by suicide and suicidal ideation (B = 0.04, *p* <.001). Conversely, ACE scores demonstrated a significant negative and indirect association with mental health help-seeking attitudes (B = − 0.01, *p* <.05). Further, suicidal ideation was significantly and negatively associated with mental health help-seeking attitudes (B = − 0.05, *p* =.01). Similarly, planning to die by suicide showed an indirect and significant negative association with mental health help-seeking attitudes (B = − 0.08, *p* =.01) (see Table 4).

## Discussion

This study’s use of intersectionality and trauma-informed frameworks is critical for understanding the complex interplay of ACEs, age, suicidal behaviors, and mental health help-seeking attitudes among Black young adults [24, 39, 40]. By integrating these frameworks, the research highlights the unique challenges faced by this demographic and emphasizes the need for culturally sensitive interventions that acknowledge the multifaceted nature of their experiences and the impact of systemic factors on mental health outcomes. Intersectionality allows for a deeper understanding of how systems of oppression like racism, ageism, and classism—interact with overlapping identities—such as race, age, gender, and socioeconomic status to shape mental health outcomes and access to care [26, 39, 41]. A trauma-informed lens recognizes that suicidal behaviors may be rooted in cumulative adversity and emotional distress stemming from early life experiences, and that healing requires safety, trust, and empowerment [24]. Findings suggest that high ACE scores may be a risk factor for suicidal behaviors, with ACE scores directly affecting suicidal ideation and suicide planning and indirectly affecting suicide attempts. Additionally, results indicate that age may serve as a protective factor for suicidal ideation among Black young adults. Further, study findings demonstrate a significant direct effect of suicidal ideation and planning to die by suicide on suicidal attempts, underscoring the importance of accessible, culturally appropriate suicide prevention and early intervention approaches. Finally, endorsement of suicidal behaviors was associated with negative attitudes toward mental health help-seeking among Black young adults, indicating the need for culturally tailored community-level interventions that address attitudes and beliefs about mental health help-seeking. These interventions must be grounded in an understanding of historical and ongoing mistrust of mental health institutions and should be designed to affirm cultural identity and promote psychological safety [24, 42–45]. 

This study’s findings are consistent with recent research documenting increasing rates of suicidal thoughts and behaviors among Black young adults [46, 47]. Among this study participants, 50% reported past-year suicidal ideation, 35% reported planning to die by suicide in the past year, and 25% reported a past-year suicide attempt. The prevalence of suicidal ideation in this sample was higher than reported in other studies. For instance, findings from the Healthy Minds Study, which surveyed over 100,000 undergraduates, indicated that 34% of Black male undergraduates faced major mental health challenges, suicidal ideation, or self-injury [47]. A more recent national sample of Black males revealed that 39% reported meeting the threshold for high risk for suicidal behaviors [47]. Moreover, the suicide rate among Black female youth aged 15–24 years increased from 2.7 to 4.3 per 100,000 between 2013 and 2019 [48, 49]. These findings underscore the severe mental health crisis among Black young adults and demonstrate the urgent need for accessible, acceptable, and culturally appropriate prevention and intervention approaches. Such approaches must be informed by intersectional and trauma-informed principles that recognize the compounding effects of racial trauma, economic hardship, and social marginalization [26, 39, 41, 45]. 

Our results demonstrate the importance of understanding Black young adults’ exposure to ACEs when assessing suicide risk and developing targeted prevention and intervention efforts. Our model indicated that ACE scores were a risk factor for suicidal thoughts and behaviors, directly affecting suicidal ideation and suicidal planning, and indirectly influencing suicidal attempts [14]. Black young adults in this sample were exposed to an average of five ACEs, indicating significant childhood trauma, which is consistent with existing literature [50, 51]. The relatively high standardized coefficients suggest a significant and positive direct association between ACE scores and both suicidal ideation (β = 0.29) and suicidal planning (β = 0.30). These findings underscore the connection between early life experiences and subsequent mental health outcomes. The significant positive indirect effect of ACE scores on suicide attempts further illustrates that ACEs may exacerbate mental health struggles, potentially increasing escalation from ideation and planning to actual attempts. A trauma-informed approach to suicide prevention must address these underlying experiences of adversity and promote healing through culturally responsive care that fosters resilience and emotional safety [40]. 

Our findings align with prior literature indicating that Black young adults who reported high ACE scores also had a greater number of suicide attempts [13, 14]. Understanding the impact of ACEs on suicidality enhances our ability to identify Black young adults at higher risk for suicidal ideation and planning early for connecting them to prevention and early intervention services. The relationship between ACEs and suicidality also highlights the importance of developing and implementing trauma-informed suicide prevention and early intervention approaches. Addressing underlying trauma exposure may mitigate the progression from suicidal ideation to planning and attempts. These efforts must be situated within an intersectional framework that considers how systemic inequities and cultural stigma shape both the experience of trauma and the pathways to care [25, 39]. 

We also found that age may serve as a protective factor among Black young adults, as it had a significant and negative direct effect on suicidal ideation. In our sample, older Black young adults in the 18-to-24-year-old range were less likely to report suicidal ideation than their younger counterparts. Although literature consistently demonstrates increasing suicidality among young adults aged 18 to 24, most studies assessing the relationship between age and suicidality have focused on children and adolescents [5, 6]. Our results suggest that targeting Black young adults aged 18 to 21 may be particularly relevant for suicide prevention and intervention efforts and that existing calls for universal and developmentally tailored suicide prevention strategies during childhood and early adolescence should be expanded to include young adults [52]. It is also important to further explore factors that may drive the negative effect of age on suicidal ideation among Black young adults. For instance, younger Black adults may experience more transitions and higher stress as they navigate next steps after high school and increased independence, while older Black young adults may have successfully navigated these transitions and feel more stable. Intersectional analysis can help illuminate how age-related stressors intersect with racial and socioeconomic pressures, shaping mental health trajectories in nuanced ways.

Further, study findings revealed a clear progression from suicidal ideation to planning and attempts, emphasizing the need for universal, culturally appropriate suicide prevention and early intervention efforts to prevent the escalation of suicidal behaviors. The results demonstrated that both suicidal ideation and suicidal planning had significant positive and direct associations with the likelihood of suicide attempts. These findings align with existing research, reinforcing the established relationship between these factors [53–55]. Understanding the relationship between suicidal behaviors among Black young adults and recognizing the increased risk of suicide attempts among those who experience suicidal ideation and plan to die by suicide supports the ability of mental health researchers and practitioners to develop specific outreach, prevention, and early intervention initiatives, as well as to strategically allocate resources to support this group. Involving community leaders, mentors, and educators in supporting young adults’ mental health is crucial. Community-based education and outreach focused on supporting Black young adults’ mental health could include approaches that encourage the disclosure of suicidal ideation and support community members’ ability to recognize warning signs for suicidality and engage in open conversations about suicidal behaviors and available resources. Additionally, community-based education and outreach could focus on disseminating suicide prevention strategies, such as safe firearm storage and lethal means safety, through trusted community organizations and services. These strategies must be trauma-informed, emphasizing empowerment, trust-building, and cultural relevance, and must be intersectional in their design to address the unique needs of Black young adults across diverse backgrounds and lived experiences.

Finally, suicidal behaviors had significantly negative direct and indirect associations with mental health help-seeking attitudes, suggesting that stigma, cultural mistrust, or structural barriers may prevent those most at risk from seeking support. Black young adults may face cultural stigma surrounding mental health issues that influence their attitudes toward help-seeking, making them less likely to seek care after a suicide attempt [56]. This stigma, and the associated attitudes about mental health help-seeking, may stem from community or cultural beliefs, fear of judgment, or a desire to handle problems independently. Mental health help-seeking attitudes may also be related to distrust of mental health systems due to historical disparities and negative experiences with healthcare providers. Scholars suggest that general mistrust of mental health professionals creates barriers to treatment access among system-involved Black girls and other populations of color [39, 56–58]. Further, Black young adults may internalize mental health struggles or prioritize other responsibilities, leading to attitudes that view mental health treatment as a secondary priority [40, 59]. Intersectionality and trauma-informed frameworks are essential for understanding these attitudes, as they reveal how cultural identity, historical trauma, and systemic exclusion converge to shape perceptions of mental health care [22, 40]., Efforts to improve help-seeking must center the voices of Black young adults and promote culturally affirming, trauma-sensitive care that builds trust and reduces stigma. Intersectionality and trauma-informed frameworks are essential for understanding these attitudes, as they reveal how cultural identity, historical trauma, and systemic exclusion converge to shape perceptions of mental health care [19, 24]. Efforts to improve help-seeking must center the voices of Black young adults and promote culturally affirming, trauma-sensitive care that builds trust and reduces stigma [39]. 

Our results suggest that further research is needed about Black young adults’ attitudes and beliefs about mental health help-seeking, with a focus on developing strategies to improve these attitudes. Additionally, it is critical to explore the cultural determinants influencing attitudes toward mental health help-seeking among Black young adults, specifically focusing on the role of cultural norms and community values in shaping the willingness to seek mental health support. Our findings also indicate the importance of community outreach efforts to destigmatize mental health care, increase awareness about the importance of seeking help, and provide accessible resources and culturally competent mental health professionals [41]. 

## Limitations

While there are advantages to using self-report data, we must also acknowledge the limitations of relying exclusively on the same for sensitive issues such as suicidal behavior and ACEs, which may lead to underreporting. This study’s sample of Black young adults in the Midwest, drawn from Qualtrics panels, may not fully represent the broader population, limiting the generalizability of our findings. The reliance on a pre-existing panel could introduce selection bias, as participants who opt into such panels may differ systematically from those who do not. Additionally, the panel’s demographics may lack the diversity present within the Black community, including variations in socioeconomic status, educational background, and cultural experiences. Lastly, the self-selection nature of panel recruitment may result in a homogeneity of perspectives, potentially overlooking the full spectrum of experiences and challenges faced by Black young adults in the region. The cross-sectional design inherently restricts our ability to draw causal inferences from the observed relationships. The associations identified between ACE scores, suicidal ideation, planning to die by suicide, suicidal attempts, and mental health help-seeking attitudes may reflect correlations rather than direct causal pathways. Longitudinal research is needed to elucidate the temporal dynamics of these variables and establish causality. Lastly, the tools or scales used to measure constructs like ACEs, suicidal ideation, and mental health help-seeking attitudes may have limitations in capturing nuanced aspects of these experiences, potentially impacting the study’s findings. Despite these limitations, the current sample enabled us to examine the prevalence and associations of ACEs among a sample of Black young adults, a population underrepresented in mental health research.

### Practice implications

Mental health practitioners can develop and implement interventions culturally relevant and sensitive to the experiences of Black young adults. Training for mental health professionals must include cultural competence and an understanding of systemic issues, such as racism and historical trauma. This will help build trust and rapport with clients, making them more likely to seek help. Establishing community-driven mental health support groups focused on shared experiences and cultural understanding can provide safe spaces for Black young adults to discuss their mental health challenges. Community leaders and organizations should be engaged to facilitate these discussions and ensure they are culturally affirming.

Integrating trauma-informed practices into mental health services can help professionals understand the impact of ACEs on Black young adults. This approach emphasizes safety, trustworthiness, peer support, and collaboration, all of which can mitigate the effects of trauma and improve treatment outcomes. Implementing outreach initiatives that specifically address the unique barriers to help-seeking behaviors among this population can help destigmatize mental health treatment. Programs can focus on educating communities about the importance of mental health and the availability of culturally competent resources.

### Policy implications

Policymakers should allocate more funds for mental health services specifically designed for Black young adults, ensuring their accessibility and capability to meet their unique needs. This includes funding for community health workers who can bridge gaps between service providers and the community. Advocacy for policies that address systemic racism and inequities in healthcare access is crucial. Policymakers should focus on reforms that reduce barriers to mental health care, such as insurance coverage for therapy and counseling services tailored for marginalized populations. Policies should mandate the inclusion of ACE screenings in routine health assessments for young adults. This can help identify at-risk individuals earlier and connect them to appropriate mental health resources before crises occur. Schools and universities should implement policies promoting mental health awareness and providing mental health resources for students. This includes training faculty to recognize signs of distress and ensuring that counseling services are culturally competent and accessible to Black young adults.

### Theoretical implications

Future research should expand intersectionality theoretical framework to include a more nuanced understanding of how interlocking systems of oppression, such as racism, sexism, classism, and heteronormativity interact to shape mental health outcomes among Black young adults [25, 26, 39, 40]. This can lead to more comprehensive models that account for the complexity of their experiences. The findings highlight the need for theoretical models that incorporate social determinants of health in understanding suicidality among Black young adults. Future research should explore how factors like socioeconomic status, community resources, and systemic inequalities intersect with mental health outcomes [19]. Theoretical frameworks should also focus on resilience among Black young adults. Understanding the protective factors that foster resilience in this population can inform the development of interventions enhancing coping strategies and overall well-being. The observed protective effect of age on suicidal ideation suggests a need for theoretical models that consider developmental trajectories in mental health. Longitudinal studies that assess the evolving relationship between age, ACEs, and mental health outcomes can provide deeper insights into how interventions can be tailored to different life stages. By addressing these practice, policy, and theoretical implications, stakeholders can work toward better mental health outcomes for Black young adults, ultimately contributing to a reduction in suicide rates and improved overall well-being.

## Conclusion

This study identified important relationships between ACE scores, age, suicidal behaviors, and mental health help-seeking attitudes among Black young adults. Our findings suggest that ACE exposures are a significant risk factor for suicidal behaviors; age may serve as a protective factor against suicidal ideation; and suicidal behaviors are both directly and indirectly associated with negative attitudes toward mental health help-seeking. These results represent an important step toward understanding the factors contributing to rising suicidality among Black young adults and identifying targets for comprehensive suicide prevention and early intervention strategies. Guided by intersectionality and trauma-informed frameworks, this study underscores the need for culturally grounded and structurally responsive approaches that reflect the lived experiences of Black young adults and address the impact of systemic inequities, historical trauma, and cultural mistrust of mental health institutions.

## Data Availability

Data supporting the findings of this study are available upon request under a license agreement. Written applications can be made to the corresponding author ([boyd.465@osu.edu](mailto: boyd.465@osu.edu)).

## References

[1] Boyd DT, Quinn CR, Durkee MI, Williams EDG, Constant A, Washington D, Butler-Barnes ST, Ewing AP. Perceived discrimination, mental health help-seeking attitudes, and suicidal ideation, planning, and attempts among Black young adults. BMC Public Health. 2024;24(1):2019. 39075376 10.1186/s12889-024-19519-1 PMC11285399

[2] Stone DM. Notes from the field: Recent changes in suicide rates, by race and ethnicity and age group—United States, 2021. MMWR. Morbidity and Mortality Weekly Report. 2023;72(6):145–6. 10.15585/mmwr.mm7206a4. 10.15585/mmwr.mm7206a4 PMC9925140 36757870

[3] Smith BL, Boyd DT, Quinn CR. Examining the impact of social connectedness on depression and suicide ideation among Black youth experiencing discrimination: a path analysis. J of Racial and Ethnic Health Disparities. 2025;12(2):1228–39. 10.1007/s40615-025-02414-9 PMC13157422 40237954

[4] Bommersbach TJ, Rosenheck RA, Rhee TG. National trends of mental health care among US adults who attempted suicide in the past 12 months. JAMA Psychiatr. 2022;79(3):219–31. 10.1001/jamapsychiatry.2021.3958 PMC8771432 35044428

[5] Goodwill JR. Reasons for suicide in black young adults: a latent class analysis. J Racial Ethn Health Disparities. 2024;11(1):425–40. 36867388 10.1007/s40615-023-01530-8 PMC9983538

[6] Twenge JM, Cooper AB, Joiner TE, Duffy ME, Binau SG. Age, period, and cohort trends in mood disorder indicators and suicide-related outcomes in a nationally representative dataset, 2005–2017. J Abnorm Psychol. 2019;128(3):185. 30869927 10.1037/abn0000410

[7] Goodwill JR, Yasui M. Mental health service utilization, school experiences, and religious involvement among a National sample of black adolescents who attempted suicide: examining within and cross-race group differences. Child Adolesc Soc Work J. 2022. 10.1007/s10560-022-00888-8.

[8] Bomysoad RN, Mersky JP, Hughes K, Felitti VJ, Moore KA, Semiz ÜB. Adverse childhood experiences and mental health conditions among adolescents. J Adolesc Health. 2020;67(6):868–70. 10.1016/j.jadohealth.2020.04.013. 32576484 10.1016/j.jadohealth.2020.04.013

[9] Kerker BD, Storfer-Isser A, Szilagyi M, Stein RE, Garner AS, O’Connor KG, Hoagwood KE, Horwitz SM. Do pediatricians ask about adverse childhood experiences in pediatric primary care? Acad Pediatr. 2016;16(2):154–60. 26530850 10.1016/j.acap.2015.08.002 PMC4779699

[10] Schilling EA, Aseltine RH, Gore S. Adverse childhood experiences and mental health in young adults: A longitudinal survey. BMC Public Health. 2007;7(30):1–10. 10.1186/1471/2458-7-30. 17343754 10.1186/1471-2458-7-30 PMC1832182

[11] Boullier M, Blair M. Adverse childhood experiences. Paediatr Child Health. 2018;28(3):132–7.

[12] Merrick MT, Ports KA, Ford DC, Afifi TO, Gershoff ET, Grogan-Kaylor A. Unpacking the impact of adverse childhood experiences on adult mental health. Child Abuse Negl. 2017;69:10–9. 28419887 10.1016/j.chiabu.2017.03.016 PMC6007802

[13] Björkenstam C, Kosidou K, Björkenstam E. Childhood adversity and risk of suicide: Cohort study of 548,721 adolescents and young adults in Sweden. BMJ. 2017;357. 10.1136/bmj.j1334. 10.1136/bmj.j1334 28424157

[14] Pournaghash-Tehrani SS, Zamanian H, Amini-Tehrani M. The impact of relational adverse childhood experiences on suicide outcomes during early and young adulthood. J Interpers Violence. 2021;36(17–18):8627–51. 31142213 10.1177/0886260519852160

[15] Bernard DL, Smith Q, Lanier P. Racial discrimination and other adverse childhood experiences as risk factors for internalizing mental health concerns among black youth. J Trauma Stress. 2022;35(2):473–83. 34800051 10.1002/jts.22760 PMC9035019

[16] Marks LR, Acuff SF, Withers AJ, MacKillop J, Murphy JG. Adverse childhood experiences, racial microaggressions, and alcohol misuse in black and white emerging adults. Psychol Addict Behav. 2021;35(3):274. 33734786 10.1037/adb0000597 PMC8084861

[17] Turner D, Wolf AJ, Barra S, Müller M, Gregório Hertz P, Huss M, et al. The association between adverse childhood experiences and mental health problems in young offenders. Eur Child Adolesc Psychiatry. 2021;30(8):1195–207. 32740721 10.1007/s00787-020-01608-2 PMC8310856

[18] Ross JM, Hope MO, Volpe VV. Intersections of racial/ethnic and religious identities on bodily well-being for black college-attending emerging adults. J Racial Ethn Health Disparities. 2024;11(3):1808–18. 37318713 10.1007/s40615-023-01653-y

[19] Assari S, Caldwell CH. Social determinants of perceived discrimination among black youth: intersection of ethnicity and gender. Children (Basel). 2018;5(2):24. 29462893 10.3390/children5020024 PMC5835993

[20] Barksdale CL, Molock SD. Perceived norms and mental health help seeking among African American college students. J Behav Health Serv Res. 2009;36:285–99. 18668368 10.1007/s11414-008-9138-y

[21] Williams ED, Lateef H, Gale A, Boyd D, Albrecht J, Paladino J, Koschmann E. Barriers to school-based mental health resource utilization among black adolescent males. Clin Soc Work J. 2023;51(3):246–61. 10.1007/s10615-023-00866-2 PMC10148625 37360754

[22] Crenshaw K. Mapping the margins: Intersectionality, identity politics, and violence against women of color. Stanford Law Rev. 1991;43(6):1241–99.

[23] Castle K, Conner K, Kaukeinen K, Tu X. Perceived racism, discrimination, and acculturation in suicidal ideation and suicide attempts among black young adults. Suicide Life-Threatening Behav. 2011;41(3):342–51. 10.1111/j.1943-278x.2011.00033.x. 10.1111/j.1943-278X.2011.00033.x PMC8378508 21535094

[24] Substance Abuse and Mental Health Services Administration. Trauma-informed care in behavioral health services. Services US Department of Health and Human Services; p. 15–4420. 24901203

[25] Procter N, Othman S, Jayasekara R, Procter A, McIntyre H, Ferguson M. The impact of trauma-informed suicide prevention approaches: a systematic review of evidence across the lifespan. Int J Ment Health Nurs. 2023;32(1):3–13. 35938946 10.1111/inm.13048

[26] Substance Abuse and Mental Health Services Administration. Intensive care coordination for children and youth with complex mental and substance use disorders: State and community profiles (SAMHSA Pub. No. PEP19-04-01-001). 2019. https://store.samhsa.gov/sites/default/files/intensive-care-youth-coordination-pep19-04-01-001.pdf.

[27] Sheftall A, Boyd R. Black youth suicidal behavior: what we know and where we go from here. 2022;107–13. 10.1007/978-3-031-06127-1_12.

[28] Jaiswal, J., & Halkitis, P. N. (2019). Towards a more inclusive and dynamic understanding of medical mistrust informed by science. Behavioral Medicine, 45(2), 79–85. https://doi.org/10.1080/08964289.2019.1619511 10.1080/08964289.2019.1619511 31343962 10.1080/08964289.2019.1619511 PMC7808310

[29] Williams DR. Addressing inequities in health and healthcare: challenges and opportunities. J Health Adm Educ.2024;40(3).

[30] Abrams, J. A., Hill, A., & Maxwell, M. (2019). Underneath the mask of the strong Black woman schema: Disentangling influences of strength and self-silencing on depressive symptoms among U.S. Black women. Sex Roles, 80(9–10), 517–526. 10.1007/s11199-018-0956-y 31086431 10.1007/s11199-018-0956-y PMC6510490

[31] King CA, Brent D, Grupp-Phelan J, Shenoi R, Page K, Mahabee-Gittens EM, Chernick LS, Melzer-Lange M, Rea M, McGuire TC, Littlefield A. Five profiles of adolescents at elevated risk for suicide attempts: differences in mental health service use. J Am Acad Child Adolesc Psychiatry. 2020;59(9):1058–68. 31830523 10.1016/j.jaac.2019.10.015 PMC7280071

[32] Watson-Singleton NN. Strong black woman schema and psychological distress: the mediating role of perceived emotional support. J Black Psychol. 2017;43(8):778–88.

[33] Boyd DT, Quinn CR, Durkee MI, Williams EDG, Constant A, Washington D, et al. Perceived discrimination, mental health help-seeking attitudes, and suicide ideation, planning, and attempts among Black young adults. BMC Public Health. 2024. 10.1186/s12889-024-19519-1. 39075376 10.1186/s12889-024-19519-1 PMC11285399

[34] Desch J, Mansuri F, Tran D, Schwartz SW, Bakour C. The association between adverse childhood experiences and depression trajectories in the ADD health study. Child Abuse Negl. 2023;137:106034. 36706612 10.1016/j.chiabu.2023.106034

[35] Felitti VJ, Anda RF, Nordenberg D, Williamson DF, Spitz AM, Edwards V, et al. Relationship of childhood abuse and household dysfunction to many of the leading causes of death in adults: the adverse childhood experiences (ACE) study. Am J Prev Med. 1998;14(4):245–58. 9635069 10.1016/s0749-3797(98)00017-8

[36] Goodwill JR, Taylor RJ, Watkins DC. Everyday discrimination, depressive symptoms, and suicide ideation among African American men. Arch Suicide Res. 2021;25(1):74–93. 31597538 10.1080/13811118.2019.1660287

[37] Hammer JH, Parent MC, Spiker DA. Mental help seeking attitudes scale (MHSAS): Development, reliability, validity, and comparison with the ATSPPH-SF and IASMHS-PO. J Couns Psychol. 2018;65(1):74. 29355346 10.1037/cou0000248 PMC10460514

[38] Suicide Prevention Resource Center. Financing suicide prevention in health care systems: Best practices and recommendations. Waltham MA. Education Development Center, Inc. 2019. Retrieved from https://zerosuicide.edc.org/resources/resource-database/financing-suicide-prevention-health-care-systems-best-practices-and.

[39] Quinn CR. Trauma, justice, and equity: using critical theories and concepts to address systemic harm among youth punishment system-involved black girls. Behav Sci. 2025;15(1):31. 39851835 10.3390/bs15010031 PMC11761597

[40] Geia L, Baird K, Bail K, et al. A unified call to action from Australian nursing and midwifery leaders: Ensuring that Black lives matter. Contemporary Nurse; 2020. p. 56. 10.1080/10376178.2020.1809107 32799620

[41] Motley DN, Victorian J, Denis K, Brooks BD. Applying an intersectionality framework to health services research. Fam Syst Health. 2023;41(4):417–24. 10.1037/fsh0000859. 38284973 10.1037/fsh0000859

[42] Lawrence HR, Burke TA, Sheehan AE, et al. Prevalence and correlates of suicidal ideation and suicide attempts in preadolescent children: a US population-based study. Transl Psychiatry. 2021;11:489. 34552053 10.1038/s41398-021-01593-3 PMC8458398

[43] American Academy of Pediatrics. Suicide: blueprint for youth suicide prevention. www.aap.org. Published February 4. 2022. Accessed 14 Jan 2023.

[44] Horowitz LM, Bridge JA, Tipton MV, et al. Implementing suicide risk screening in a pediatric primary care setting: from research to practice. Acad Pediatr. 2022;22:217–26. 35248306 10.1016/j.acap.2021.10.012 PMC8908796

[45] Diaz AD. Assessment of suicide risk and cultural considerations in forcibly displaced migrant youth. Acad Pediatr. 2024;24(5):25–31. 38991798 10.1016/j.acap.2023.05.024

[46] Lipson SK, Kern A, Eisenberg D, Breland-Noble A. Mental health disparities among college students of color. J Adolesc Health. 2018;63(3):348–56. 10.1016/j.jadohealth.2018.04.014. 30237000 10.1016/j.jadohealth.2018.04.014

[47] Boyd DT, Durkee MI, Brown DW, Constant AS. Authenticity, Racial discrimination, depression, and suicidal ideation among black young men, united States, 2024. Am J Public Health. 2025;115(9):1417–25. 40768697 10.2105/AJPH.2025.308148 PMC12332324

[48] Ramchand R, Gordon JA, Pearson JL. Trends in suicide rates by race and ethnicity in the United States. JAMA network open. 2021;4(5):e2111563. 34037735 10.1001/jamanetworkopen.2021.11563 PMC8155821

[49] Joseph VA, Martínez-Alés G, Olfson M, Shaman J, Gould MS, Gimbrone C, Keyes KM. Trends in suicide among Black women in the United States, 1999–2020. American J Psychiatr. 2023;180(12):914–7. 10.1176/appi.ajp.20230254 PMC11205256 38037401

[50] Ports KA, Tang S, Treves-Kagan S, Rostad W. Breaking the cycle of adverse childhood experiences (ACEs): economic position moderates the relationship between mother and child ACE scores among black and Hispanic families. Child Youth Serv Rev. 2021;127:106067. 35125581 10.1016/j.childyouth.2021.106067 PMC8815463

[51] Sheftall AH, Asti L, Horowitz LM, Felts A, Fontanella CA, Campo JV, et al. Suicide in elementary school-aged children and early adolescents. Pediatrics. 2016;138(4):e20160436. 27647716 10.1542/peds.2016-0436 PMC5051205

[52] Bridge JA, Asti L, Horowitz LM, Greenhouse JB, Fontanella CA, Sheftall AH, Kelleher KJ, Campo JV. Suicide trends among elementary school-aged children in the united States from 1993 to 2012. JAMA Pediatr. 2015;169(7):673–7. 25984947 10.1001/jamapediatrics.2015.0465

[53] Lindsey MA, Sheftall AH, Xiao Y, Joe S. Trends of suicidal behaviors among high school students in the United States: 1991–2017. Pediatrics. 2019;144(5):e20191187. 31611338 10.1542/peds.2019-1187 PMC7299440

[54] Sheftall AH, Vakil F, Ruch DA, Boyd RC, Lindsey MA, Bridge JA. Black youth suicide: investigation of current trends and precipitating circumstances. J Am Acad Child Adolesc Psychiatry. 2022;61(5):662–75. 10.1016/j.jaac.2021.07.821. 34509592 10.1016/j.jaac.2021.08.021 PMC8904650

[55] Betancourt JR, Green AR, Carrillo JE, Ananeh-Firempong O, I. I. Defining cultural competence: A practical framework for addressing racial/ethnic disparities in health and health care. Public Health Rep. 2016;118(4):293–302. 10.1016/S0033-3549(04)50253-4 PMC1497553 12815076

[56] Hockenberry S, Puzzanchera C. Juvenile court statistics 2014. National Center for Juvenile Justice.2017. http://www.ncjj.org/pdf/jcsreports/jcs2014.pdf . Accessed 3 Feb 2019.

[57] Lindsey MA, Chambers K, Pohle C, Beall P, Lucksted A. Understanding the behavioral determinants of mental health service use by urban, under-resourced black youth: adolescent and caregiver perspectives. J Child Fam Stud. 2013;22(1):107–21. 23355768 10.1007/s10826-012-9668-z PMC3551580

[58] Busby DR, Zheng K, Eisenberg D, Albucher RC, Favorite T, Coryell W, et al. Black college students at elevated risk for suicide: barriers to mental health service utilization. J Am Coll Health. 2021;69(3):308–14. 31662044 10.1080/07448481.2019.1674316

[59] Early KE, Akers RL. It’s a white thing: an exploration of beliefs about suicide in the African-American community. Deviant Behav. 1993;14(4):277–96.

